# Diet in Disease

**Published:** 1893-02-25

**Authors:** Ernest Hart

**Affiliations:** Bachelier-ès-Sciences-es-Lettres (rèstreint), formerly Student of the Faculty of Medicine of Paris, and of the London School of Medicine for Women


### DIET IN DISEASE.

BY MRS. ERNEST HART, Bachelier-ès-Sciences-es-Lettres (rèstreint), formerly Student of the Faculty of Medicine of Paris, and of the London School of Medicine for Women.

### XIII.—DIABETES (*continued*).

**The Treatment of Diabetes** is one of intelligent watchfulness. Cases differ so much from one another, that no hard and fast rule applicable to all, can be laid down. What is harmful to one patient is well borne by another, and whereas a rigid regimen can be followed by one person, its maintenance leads to adverse complications in another. The successful treatment of the diabetic patient lies in fact between his cook and his doctor; in the careful and intelligent preparation of his food on the one hand by the cook, and on the other in the checking and control of his diet by the physician, according to the physical signs given by examination of the patient and his urine. On these depend the maintenance of a fair standard of health and comfort.

The following table showing the percentage composition of various articles of food will be found to be most valuable to refer to in preparing the diet not only of diabetics, but of other invalids :—

| | Water. | Albumen, &c. | Starch, &c. | Sugar. | Fat. | Salts. |
|---|---|---|---|---|---|---|
| Bread | 37· | 8·1 | 47·4 | 3·6 | 1·6 | 2·3 |
| Biscuit† | 8· | 15·6 | 73·4 | | 1·3 | 1·7 |
| Wheat flour | 15· | 10·8 | 66·3 | 4·2 | 2·0 | 1·7 |
| Barley meal | 15· | 6·3 | 69·4 | 4·9 | 2·4 | 2·0 |
| Oatmeal | 15· | 12·6 | 58·4 | 5·4 | 5·6 | 3·0 |
| Rye meal | 15· | 8·0 | 69·5 | 3·7 | 2·0 | 1·8 |
| Indian corn meal | 14· | 11·1 | 64·7 | 0·4 | 8·1 | 1·7 |
| Rice | 13· | 6·3 | 79·1 | 0·4 | 0·7 | 0·5 |
| Peas | 15· | 23·0 | 55·4 | 2·0 | 2·1 | 2·5 |
| Arrowroot | 18· | — | 82·0 | — | — | — |
| Potatoes | 75· | 2·1 | 18·8 | 3·2 | 0·2 | 0·7 |
| Carrots | 83· | 1·3 | 8·4 | 6·1 | 0·2 | 1·0 |
| Parsnips | 82· | 1·1 | 9·6 | 5·8 | 0·5 | 1·0 |
| Turnips | 91· | 1·2 | 5·1 | 2·1 | — | 0·6 |
| (1) Cabbage† | 91· | 2·0 | 5·8 | | 0·5 | 0·7 |
| Sugar | 5· | — | — | 95·0 | — | — |
| Treacle | 23· | — | — | 77·0 | — | — |
| New milk | 86· | 4·1 | — | 5·2 | 3·9 | 0·8 |
| Cream | 66· | 2·7 | — | 2·8 | 26·7 | 1·8 |
| Skim milk | 88· | 4·0 | — | 5·4 | 1·8 | 0·8 |
| Butter milk | 88· | 4·1 | — | 6·4 | 0·7 | 0·8 |
| Cheese† | 36·8 | 33·5 | — | — | 24·3 | 5·4 |
| Cheddar cheese | 36· | 28·4 | — | — | 31·1 | 4·5 |
| Skim cheese | 44· | 44·8 | — | — | 6·3 | 4·9 |
| Lean beef | 72· | 19·3 | — | — | 3·6 | 5·1 |
| Fat beef | 51· | 14·8 | — | — | 29·8 | 4·4 |
| Lean mutton | 72· | 18·3 | — | — | 4·9 | 4·8 |
| Fat mutton | 53· | 12·4 | — | — | 31·1 | 3·5 |
| Veal | 63· | 16·5 | — | — | 15·8 | 4·7 |
| Fat pork | 39· | 9·8 | — | — | 48·9 | 2·3 |
| Green bacon | 24· | 7·1 | — | — | 66·8 | 2·1 |
| Dried bacon | 15· | 8·8 | — | — | 73·3 | 2·9 |
| Ox liver | 74· | 18·9 | — | — | 4·1 | 3·0 |
| Tripe | 68· | 13·2 | — | — | 16·4 | 2.4 |
| Cooked meat, roast, no dripping being lost. Boiled, assumed to be the same | 54· | 27·6 | — | — | 15·45 | 2·95 |
| Poultry | 74· | 8·8 | — | — | 3·8 | 1·2 |
| White fish | 78· | 18·1 | — | — | 2·9 | 1·0 |
| Eels | 75· | 9·9 | — | — | 13·8 | 1·3 |
| Salmon | 77· | 16·1 | — | — | 5·5 | 1·4 |
| Entire egg | 74· | 14·0 | — | — | 10·5 | 1·5 |
| White of egg | 73· | 20·4 | — | — | — | 1·6 |
| Yolk of egg | 52· | 16·0 | — | — | 30·7 | 1·3 |
| Butter and fats | 15· | 0·1 | — | — | 83·0 | 2·0 |
| Beer and porter | 91· | 0·1 | — | 8·7 | — | 0·2 |

There is one broad rule to be followed in the preparation of the diet of a diabetic, that is to avoid all articles containing starch and sugar. The following list of articles of diet which may be allowed to or must be forbidden a diabetic, is based both on the published opinions of observers and on practical experience, and is given as a dietary table which may be faithfully followed.

| *Allowed.* | *Forbidden.* |
|---|---|
| Butchers' meat of all kinds. | Sugar in any form. |
| Ham, bacon, and tongue when not sugar-cured. | Wheaten bread, oatmeal cakes, porridge. |
| Poultry and game. | Ordinary biscuits. |
| Fish of all kinds. | Rice, arrowroot. |
| Oysters and shellfish. | Potatoes, carrots. |
| Crabs, lobsters. | Parsnips, beans, and peas. |
| Beef-tea, broth, not thickened. | Sago, tapioca. |
| Soups, made of meat stock without any starchy thickening. | Macaroni, vermicelli. |
| Jellies made without sugar. | Spanish onions. |
| Aspic. | All sweet fruits, such as grapes, cherries, peaches, strawberries, apricots, plums, gooseberries, currants, oranges, and all preserved fruits. |
| Tripe. | Pastry. |
| German sausage. | Puddings of every kind which contain sugar or farinaceous foods. |
| Eggs, cheese, cream-cheese, and cream. | Beetroot. |
| Butter, fat, oil, and lard. | Liver. |
| Caviare. | English sausages. |
| Almond cakes, bran cakes and gluten bread, as substitutes for wheaten bread. | Treacle. |
| "Torrefied" or charred bread. | |
| Saccharin to replace sugar. | |
| Cabbage, endive, spinach. | |
| Broccoli, Brussels sprouts. | |
| Lettuce, spring onions. | |
| Cucumber, green asparagus. | |
| Watercress, sorrel. | |
| Salad, celery, tomatoes. | |
| Artichokes, mushrooms. | |
| Cauliflowers, sea-kale. | |
| Turnips, French beans. | |
| Vegetable marrow, dandelion. | |
| Cardoons, mustard and cress. | |
| Radishes, turnip-tops, and nettles. | |
| Unripe fruits, such as green gooseberries, green currants, and unripe apples cooked with saccharin. | |
| Nuts of all kinds except chestnuts. | |
| Sardines in oil. | |
| Foie gras. | |
| Norwegian herrings in oil. | |
| Pilchards in oil. | |
| Pickles. | |
| Savoury jelly. | |
| Custard. | |

BEVERAGES.

| *Allowed.* | *Forbidden.* |
|---|---|
| Tea and coffee. | Champagne, and all sweet and sparkling wines. |
| Cocoa made from nibs and mixed with cream. | Sweet ales, mild and old porter and stout. |
| Water, soda-water. | Cider and perry. |
| Vichy and Apollinaris waters. | Sweetened lemonade. |
| Claret and Burgundy. | Port wine and Madeira. |
| Dry Sauterne and Chablis. | Liqueurs. |
| Brandy and whisky, in small quantities, unsweetened. | Rum and sweetened gin. |
| Milk, sparingly. | Patent cocoas and chocolates. |
| Lemonade, made of fresh lemons, and sweetened with saccharin. | Sorbets. |
| Bitter ale, in moderate quantity. | Fruit juices and syrups. |
| | Ginger beer. |

From this list it will be seen that the number of articles permitted a diabetic is large, but the exclusion of certain articles which we have come in modern times to look upon as of absolute daily necessity is often felt severely, particularly at first, till the ingenuity of the cook has been exercised to discover an agreeable and varied dietary within the limits imposed. The strict exclusion of bread, the staff of life, is that which is felt most, and the patient ill reconciles himself to the gluten and bran breads and almond cakes which have been introduced as substitutes for wheaten bread, though these are useful in making an agreeable variation in the diet. It is well and wise so cleverly to design and arrange the food of a diabetic patient that he can take his meals with his family at a common table and from the same dishes, unaware, and without being constantly reminded, that he must not take this and must not touch that. To show how easily this can be done will be the object of subsequent chapters.

**The Examination of the Urine.**—In order satisfactorily to carry out the dietetic treatment of a diabetic patient, it is necessary to examine daily the urine. The initial physical sign of diabetes is the excretion of sugar; stop this, and the symptoms of thirst, dryness of the mouth, lassitude, and weakness are arrested. The effect of excluding starch and sugar from the diet can only be accurately ascertained by discovering if the excretion of sugar by the urine is arrested or diminished. There are various ways of testing the presence of sugar in the urine. The following is the easiest, and is one which can be well practised by a nurse. To a small quantity of urine in a test tube add half the amount of liquor potassæ or liquor sodæ and boil. If sugar is present a yellowish brown colour soon makes its appearance, which becomes more intense as the boiling is continued, and which will be the deeper the larger the proportion of sugar, becoming finally almost black if the quantity is very large. The coloration is produced by turning the colourless sugar into brown molasses by heat, much in the same way as a deep brown sticky fluid is produced when a piece of sugar is burnt in a candle. If now to the coloured fluid in the test tube be added a few drops of nitric acid, the brown coloration disappears, and there is an odour of burnt molasses. To ascertain the amount of sugar present is a more difficult laboratory process, but any nurse who has charge of a diabetic patient, or anybody who is closely watching the effect of diet on a diabetic person, should once a week or at least once a month collect the total quantity of urine passed in the twenty-four hours, and send a specimen to a doctor or chemist for analysis and report. This analysis will be her chart, showing her how to direct her course of dietetic treatment. For her compass she needs to take daily the specific gravity of the urine, and to ascertain roughly, though fairly correctly, the amount of urine daily passed. As it would be troublesome and inadvisable for the patient to collect the whole of the urine passed in the twenty-four hours, a sufficiently accurate estimate can be obtained by collecting and measuring the amount of urine passed at night in the bed-room. This should be measured every morning, and the specific gravity taken. The record should be entered in a book, and kept for comparison with the record of the dietary of the patient, so that the effect of the food taken can be judged by watching its influence on the amount and the specific gravity of urine excreted. The normal specific gravity of the urine is about 1·025. This varies greatly, even in healthy persons, and may rise to 1·030 or fall to 1·015 without any deviation from health, but if the specific gravity is found to be constantly at or above 1.030 sugar may be suspected. The normal amount of urine excreted by a healthy man is about three pints; in the diabetic this may be very much increased. The following extracts from the record of a diabetic patient, which was kept for several years, will show exactly what I mean, and how a nurse or non-scientific person may easily check and ascertain the effects of diet on the patient under observation.

| Date. | Quantity passed in Night. | S. G. | Remarks. |
|---|---|---|---|
| Jan. 9th ... | 30 oz. ... | 1·025 | |
| ,, 10th ... | 30 ,, ... | 1·034 | |
| ,, 11th ... | 21 ,, ... | 1·023 | |
| ,, 12th ... | 24 ,, ... | 1·031 | Dinner party, took sweets. |
| ,, 13th ... | 34 ,, ... | 1·020 | |
| ,, 14th ... | 26 ,, ... | 1·013 | Nervous, low spirits. |
| ,, 15th ... | 28 ,, ... | 1·030 | Dined out. |
| ,, 16th ... | 16 ,, ... | 1·039 | Tongue raw. |
| ,, 17th ... | 22 ,, ... | 1·030 | |
| ,, 18th ... | 22 ,, ... | 1·015 | Much better, tongue better. |
| Feb. 17th ... | 20 ,, ... | 1·025 | |
| ,, 23rd ... | — ... | 1·020 | |
| ,, 24th ... | — ... | 1·035 | Two helpings of treacle pudding. |
| ,, 26th ... | — ... | 1·028 | |
| Apr. 5th ... | 28 ,, ... | 1·030 | |
| ,, 6th ... | 30 ,, ... | 1·040 | |
| ,, 7th ... | 30 ,, ... | 1·040 | Left off taking bread. |
| ,, 8th ... | 24 ,, ... | 1·038 | |
| ,, 9th ... | 24 ,, ... | 1·033 | |
| ,, 10th ... | 29 ,, ... | 1·012 | |
| ,, 11th ... | 24 ,, ... | 1·026 | |

In this patient, who for ten years was carefully and successfully dieted, any indiscretion such as is committed when dining out, or in taking treacle pudding, was immediately detected by the specific gravity bulb, which, it will be found, gives sure and faithful directions as to the course of dieting to be pursued.

**Weighing the Patient.**—Emaciation is frequently a marked symptom of diabetes and one which it is most important to check. A weighing-machine should form part of the furniture of the bedroom of a diabetic, and he should be weighed at regular intervals and the record kept. By depriving the patient of all starch and sugar in his diet, which, as we have shown in the earlier articles, are the foods out of which much of the fat of the body is manufactured, and by his wasteful excretion of sugar, the body naturally emaciates. It becomes, therefore, of the greatest possible importance when framing his dietary not only to exclude starch and sugar, but to compensate for this exclusion by giving an excess of fatty foods. The influence of these must be noted by means of the weighing machine.

We thus see it is not only necessary to diet a diabetic patient, but closely and carefully to watch the effect of this diet on him, by ascertaining and recording its influence on the specific gravity and amount of urine daily secreted, and on his loss or gain of flesh.

